# Preparation of Waste PP/Fly Ash/Waste Stone Powder Composites and Evaluation of Their Mechanical Properties

**DOI:** 10.3390/ma16103687

**Published:** 2023-05-12

**Authors:** Jun Cong Ge, Eun Seo Lee, Deuk Ju Kim, Ji Ho Kang, Ik Tae Im, Nag Jung Choi

**Affiliations:** 1 Division of Mechanical Design Engineering, Jeonbuk National University, Jeonju-si 54896, Republic of Korea; jcge@jbnu.ac.kr (J.C.G.); itim@jbnu.ac.kr (I.T.I.); 2 Art Stone Co., Ltd., 64, Howondae 3-gil, Impi-myeon, Gunsan-si 54058, Republic of Korea; lhk000330@naver.com (E.S.L.); artstonek@naver.com (D.J.K.); jhkang@kunjang.ac.kr (J.H.K.)

**Keywords:** waste recycling, polypropylene, fly ash, waste stone powder, mechanical properties

## Abstract

The research was carried out to analyze the combined and mechanical properties of polypropylene (PP)/fly ash (FA)/waste stone powder (WSP) composite materials. PP, FA and WSP were mixed and prepared into PP100 (pure PP), PP90 (90 wt% PP + 5 wt% FA + 5 wt% WSP), PP80 (80 wt% PP + 10 wt% FA + 10 wt% WSP), PP70 (70 wt% PP + 15 wt% FA + 15 wt% WSP), PP60 (60 wt% PP + 20 wt% FA + 20 wt% WSP) and PP50 (50 wt% PP + 25 wt% FA + 25 wt% WSP) composite materials using an injection molding machine. The research results indicate that all PP/FA/WSP composite materials can be prepared through the injection molding process and there are no cracks or fractures found on the surface of the composite materials. The research results of thermogravimetric analysis are consistent with expectations, indicating that the preparation method of the composite materials in this study is reliable. Although the addition of FA and WSP powder cannot increase the tensile strength, it is very helpful to improve the bending strength and notched impact energy. Especially for notched impact energy, the addition of FA and WSP results in an increase in the notched impact energy of all PP/FA/WSP composite materials by 14.58–22.22%. This study provides a new direction for the reuse of various waste resources. Moreover, based on the excellent bending strength and notched impact energy, the PP/FA/WSP composite materials have great application potential in the composite plastic industry, artificial stone, floor tiles and other industries in the future.

## 1. Introduction

Because of their light weight, good toughness and strength, plastics are widely used in plastic bags, packaging, automotive vehicles, electrical appliances, mobile phones and other electronic products [1]. However, plastic (a synthetic resin) is mainly made from fossil oil and gas through polymerization, polyaddition, polycondensation, addition-condensation and other polymerization reactions. It has a long life cycle and strong decomposition resistance [2,3]. With the widespread use of plastics, the pollution caused by waste plastics is gradually threatening the ecological environment and human health. According to relevant data, as of 2016, about 9 to 23 million metric tons of waste plastics per year were discharged into rivers, lakes and oceans and about 13 to 25 million metric tons per year were discharged into the land environment. If not regulated and treated, the discharge of waste plastics is expected to be twice as much as that of 2016 by 2025 [4].

Plastics have been widely used in daily life due to their advantages and together with steel and cement, they entered the top three in the world of the most widely manufactured materials during the COVID-19 pandemic [5]. According to reports, approximately 8–11 million tons of waste plastic are discharged into the ocean every year, which will cause serious harm to marine life [6]. Leslie et al. [7] developed a reliable and sensitive dual-radiation pyrolysis sampling and analysis method and applied it to the detection and analysis of plastic particles (≥700 nm) in the whole blood of 22 healthy volunteers. They found microplastics in human blood for the first time and these microplastics may also enter human organs. Although the way in which microplastics enter the human body has not been fully clarified, it is likely to be carried out mainly through direct or indirect ingestion. If microplastics carrying heavy metals enter the human body, they will cause great harm to the human body [8]. Therefore, it is of great significance to develop the recycling technology for waste plastics and improve their recycling rate to reduce environmental pollution and achieve the goal of global carbon neutrality as soon as possible.

Generally, the recycling methods of waste plastics are mainly divided into three types: energy/thermal recycling, physical/mechanical recycling and chemical recycling [9,10]. Energy recycling refers to the use of plastic waste as an alternative fuel for boilers, generators and diesel engines [11]. Physical recycling refers to the process of transforming waste plastics into recycled raw materials (pellets) through mechanical crushing, screening and separation, extrusion molding and other processes [10]. Finally, chemical recycling means that the waste plastics (polymer) are completely restored to the original materials (monomer) through some chemical reactions (e.g., solvolysis, pyrolysis and gasification) [12]. The most widely used plastic polymers include polypropylene (PP), polyethylene (PE), polyethylene terephthalate (PET), polystyrene (PS), polyurethane (PU) and polyvinyl chloride (PVC), which are also the components of major urban plastic waste [13]. Among them, PP and PE are the most widely used plastics. Compared to PE, PP generally has low density, low price, high hardness and high tensile strength [14]. In addition, PP also has good heat resistance, acid and alkali corrosion resistance and good mechanical properties, so it is widely used in the field of food packaging. Moreover, PP is a semicrystalline plastic, which has greater high temperature resistance, fatigue resistance and chemical properties compared to amorphous plastic [15]. Therefore, PP was selected as the key research object in this study.

On the other hand, as industrial wastes, fly ash (FA) and waste stone powder (WSP) have also received great attention recently for their reuse. FA refers to the ash collected through the dust collector when pulverized coal is burned in a thermal power plant. Ash that is not collected by the dust collector after combustion is called bottom ash (BA). A total of 80% of the ash generated in coal-fired power generation is FA. FA is mainly composed of spherical particles with different diameters (mostly microns), which are divided by the chemical composition into aluminosilicate and iron-containing. The aluminosilicate spherical particles are mainly composed of SiO_2_ (41.82~61.27%), Al_2_O_3_ (17.03~22.8%), FeO and Fe_2_O_3_ (~8.31%), MgO (~4.83%), K_2_O (~3.05%), TiO_2_ (~1.04%) and Na_2_O (~0.93%) [16]. As the main energy of thermal power generation, coal supplied about 30% of primary energy and 41% of global power generation in 2012. In 2012, the global coal ash output exceeded 800 million tons and is expected to exceed 13.3 billion tons by 2023 [17]. The large amount of FA will cause serious environmental pollution problems (air, water and soil pollution) and social problems (occupying a large amount of land area) [18]. Based on its unique physical and chemical properties, FA is widely used in additives of cement and concrete products, structural filling and covering materials, lightweight aggregate additives, building materials, synthetic zeolite, water and soil improvers [19]. However, at present, the global average utilization rate of FA has not exceeded 60% and a large number of FA is still directly discharged into the air or buried. Another industrial waste, WSP, is the powder produced during stone processing. Its main composition is similar to that of FA and also mainly consists of SiO_2_ (50~70%) and Al_2_O_3_ (10~30%). The waste stone and WSP produced in the process of granite collection or processing are only recycled as part of recycled aggregate and the rest are mostly illegally landfilled or discarded without authorization.

Therefore, in order to improve the reuse rate of various industrial wastes (waste PP, FA and WSP) and study the mechanical properties of PP/FA/WSP composite materials, PP, FA and WSP were first prepared into composite plastic pellets by means of physical recycling technology using an extruder machine according to a certain mixing ratio and then these composite plastic pellets with different mixing ratios were prepared into various PP/FA/WSP composite materials using an injection molding machine. The characteristics of these composite materials were investigated through various chemical analysis methods such as SEM, EDS, XRD and FTIR. The mechanical properties including tensile strength, bending strength and notched impact energy were compared and analyzed. This study provides a valuable reference for reducing solid waste and improving the utilization rate of various industrial wastes. The PP/FA/WSP composite materials with excellent bending strength and notched impact energy have great potential application value in the construction industry such as artificial synthetic stone, synthetic plastics, automobile industry, floor tiles and other building industries.

## 2. Materials and Methods

### 2.1. Preparation of Composite Plastic Pellets

**Figure 1:**
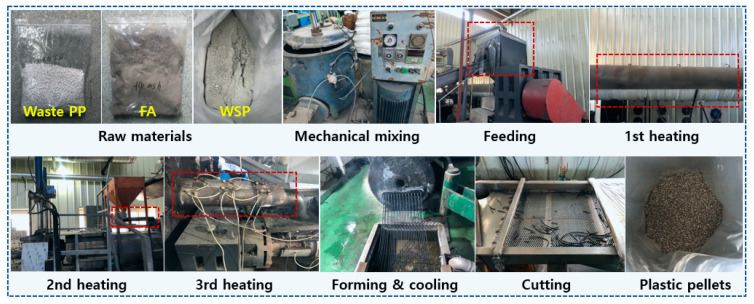
Process flow for preparation of PP/FA/WSP composite pellets.

In this study, waste PP, FA and WSP were purchased from the companies Yongsan Chemical Co., Ltd. (Gimje-si, Jeollabuk-do, Republic of Korea), K-hana Cement Co., Ltd. (Gunsan-si, Jeollabuk-do, Republic of Korea) and Art Stone Co., Ltd. (Gunsan-si, Jeollabuk-do, Republic of Korea), respectively. Firstly, PP, FA and WSP were mechanically mixed for 30 min by a mass ratio of 1:0.5:0.5 and then the mixed sample was subjected to feeding, first heating, second heating, third heating, molding and cooling, and cutting to obtain the final PP/FA/WSP composite plastic pellets. The specific preparation process is shown in Figure 1.

### 2.2. Injection Molding Experimental Details

**Figure 2:**
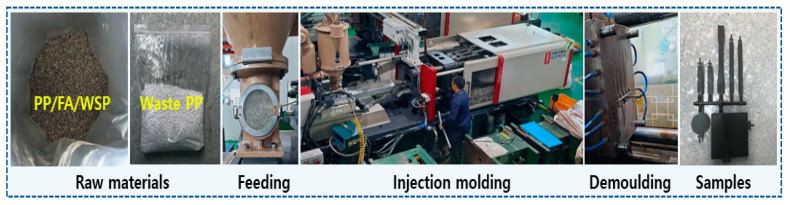
Process flow for preparation of PP/FA/WSP composite samples.

The PP/FA/WSP composite plastic pellets were mixed with pure waste PP in the mass ratios of 0:20, 4:16, 8:12, 12:8, 16:4, 20:0 for 30 min, which were denoted as PP100, PP90, PP80, PP70, PP60 and PP50, respectively. These mixed samples were then prepared into various PP/FA/WSP composite materials using a 170-ton injection molding machine provided by Heejin Co., Ltd. (Gimje-si, Jeollabuk-do, Republic of Korea). The diameter of the screw was 25 mm, the injection pressure was 20 MPa, the mold temperature was 230 °C, the cylinder temperature was 34 °C and the molding rate was 5/s. The injection molding process is shown in Figure 2.

### 2.3. Characterization Analysis Method

The surface structure and composition of the injection molded PP/FA/WSP composite materials were investigated using the field emission scanning electron microscopes (FE-SEM) and energy-dispersive X-ray spectroscopy (EDS) (SUPRA40VP, Carl Zeiss, Jena, Germany), respectively. A high-resolution X-ray diffractometer (HR-XRD, Empyrean, Panalytical, Malvern, UK) was used to analyze the crystal structure and orientation of PP/FA/WSP composite materials in a Panalytical MRD Pro system using Cu Kα1 radiation (λ = 1.54 nm). The chemical structure changes were investigated using the Fourier transform infrared spectrometer (FT-IR, Spectrum 3, Perkin Elmer, Beaconsfield, UK) in the region of 4000–400 cm^−1^ with a resolution of 1 cm^−1^.

### 2.4. Thermogravimetric Analysis

Thermogravimetric analysis (TGA) is an ideal method for evaluating the thermal stability of various materials and their mixtures. In this study, approximately 10 mg of samples were cut from PP/FA/WSP composite materials for TGA analysis under an N_2_ atmosphere at a heating rate of 10 °C/min from room temperature to 700 °C. A TGA analyzer (Discovery SDT 650, TA Instrument, New Castle, DE, USA) was employed to analyze the thermal decomposition characteristics.

### 2.5. Mechanical Properties Analysis Method

**Figure 3:**
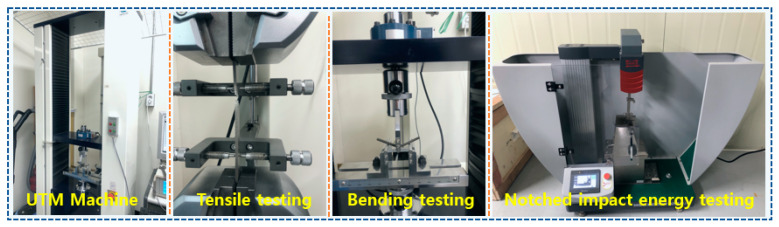
Experimental equipment for testing mechanical properties.

In this study, the typical mechanical properties including tensile strength, bending strength and notched impact energy were compared and investigated. The tensile strength and three-point bending test were carried out on a KDPI-130 Series Universal Testing Machine (UTM, KD Precision, Seoul, Republic of Korea) with a 100 kN load cell according to ASTM D638 and ASTM D790, respectively. The notched Izod impact measurements were carried out using a pendulum impact tester (PIT-J, Shenzhen Wance Testing Machine Co., Ltd., Shenzhen, China) with a nominal energy capacity of 2 J according to ASTM D256. The equipment used in the experiment is shown in Figure 3. According to ASTM test standards, each mechanical property was tested 5 times and the average value was calculated as the final experimental data.

## 3. Results and Discussion

### 3.1. Characterization Analysis

#### 3.1.1. SEM and EDS Analysis

**Figure 4:**
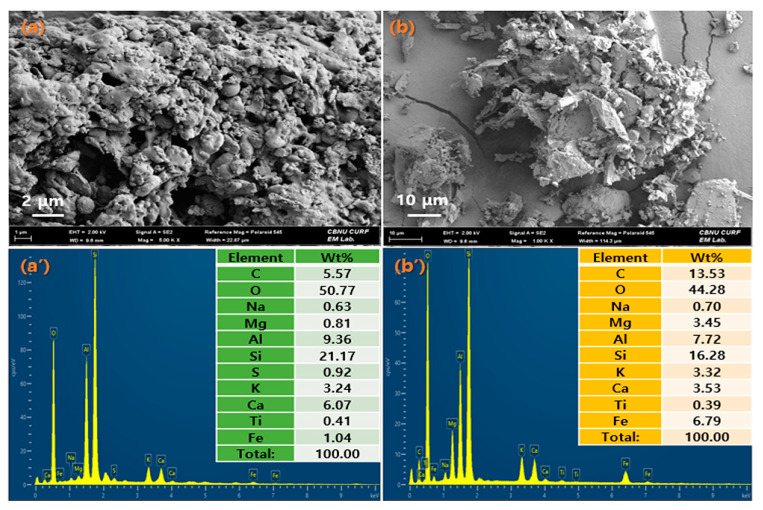
SEM and EDS for (**a**,**a**’) fly ash and (**b**,**b**’) waste stone powder.

Figure 4 shows the SEM and EDS analysis of fly ash (FA) and waste stone powder (WSP), respectively. The SEM structure shows that FA is composed of spherical particles with different sizes, while WSP is composed of particles in the form of blocks and strips. This is consistent with the reports of [20,21]. The diameter of FA particles is significantly smaller than that of WSP, which is more conducive to mixing with other organic or inorganic substances. On the other hand, the EDS analysis shows that FA is composed of elements such as C, O, Na, Mg, Al, Si, S, K, Ca, Ti and Fe, which accounts for 5.57, 50.77, 0.63, 0.81, 9.36, 21.17, 0.92, 3.24, 6.07, 0.41 and 1.04 wt%, respectively; WSP is composed of C, O, Na, Mg, Al, Si, K, Ca, Ti and Fe, which accounts for 13.53, 44.28, 0.70, 3.45, 7.72, 16.28, 3.32, 3.53, 0.39 and 6.79 wt%, respectively. Except for the fact that WSP does not contain S element, the remaining components are the same as those of FA. In addition, both FA and WSP contain high Si elements, which provide rich raw materials for the preparation of hard materials. Moreover, the presence of Ca element also provides the possibility of improving crystallization in hydration reactions, so both FA and WSP can be used as raw materials for cement substitute material [22,23]. Other metal elements such as Al, Fe and Mg can also be extracted and used as other metal minerals. The existence of a large number of valuable metal and non-metallic elements means that FA and WSP have great recycling value. The reuse of FA and WSP can develop a variety of new products with high added value, which not only helps to reduce environmental pollution but also has important significance in improving the reuse rate of waste.

**Figure 5:**
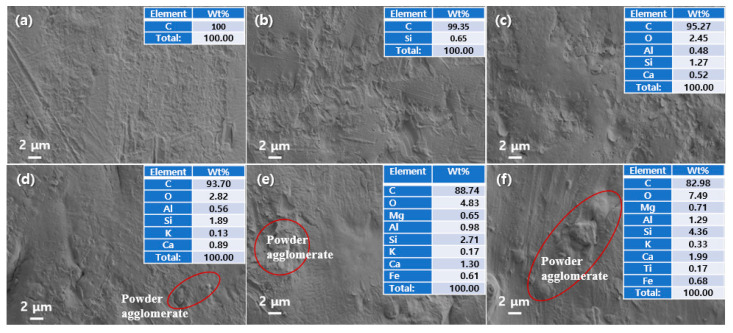
SEM and EDS for (**a**) PP100, (**b**) PP90, (**c**) PP80, (**d**) PP70, (**e**) PP60 and (**f**) PP50.

Figure 5 shows the SEM and EDS analysis of PP/FA/WSP composite materials. From the SEM images, it can be seen that PP100 has a relatively smooth surface compared to other samples and some particles or particle aggregation phenomena begin to appear on the composite materials with the addition of FA and WSP (seen red area in Figure 5d–f). In addition, there are no cracks, fractures or other defects on the surface of PP/FA/WSP composites. This indicates that this injection molding process is feasible, even if the total addition of FA and WSP reaches 50 wt%. On the other hand, the EDS results show that PP100 is only composed of C element, while all composites contain Si element. By increasing the amount of FA and WSP added, the content of Si element presents a gradually increasing trend. In Figure 5b–d, the number of elements detected is lower than in Figure 5e,f, which may be due to the lower addition of FA and WSP, both lower than 30 wt%. However, with the addition of FA and WSP, the C element presents a gradually decreasing trend, while other elements present an opposing trend. This indicates that PP, FA and WSP are fully mixed during the preparation of PP/FA/WSP composites and their distribution is relatively uniform. Adding high mixing ratios of FA and WSP has no negative impact on injection molded composites and its successful mixing ratio provides an important data reference value for processing other products.

#### 3.1.2. XRD and FT-IR Analysis

**Figure 6:**
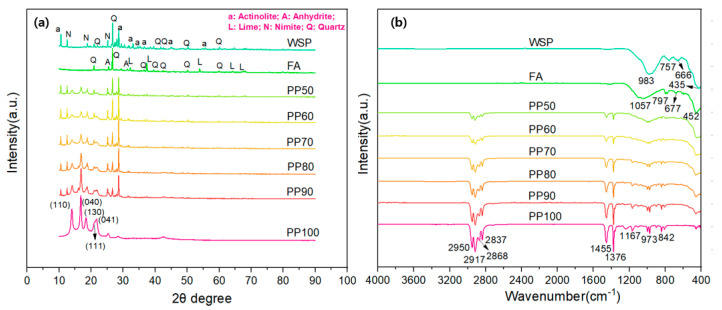
XRD pattern (**a**) and FTIR spectra (**b**).

Figure 6 shows the XRD and FTIR spectra of FA, WSP and PP/FA/WSP composite materials. XRD is a typical analytical tool for investigating crystal phases present in substances, which determines a certain crystal phase based on the diffraction angle of X-ray beams scattered at different positions of atoms in the crystal. In Figure 6a, the XRD result shows that the pure PP sample has five relatively obvious peaks, mainly appearing between 10 and 25 degrees (2θ). These peaks correspond to the crystalline phase of the isotactic polypropylene located at 14.19°, 17.05°, 18.65°, 21.36° and 21.87°, which correspond to the (1 1 0), (0 4 0), (1 3 0), (1 1 1) and (0 4 1) diffraction planes, respectively. These peaks are typical α-form crystallographic plane of PP [24,25]. With the decrease in PP content, the strength of these five typical peaks in the PP/FA/WSP composite materials is gradually weakened, which is mainly related to the decrease in PP content. In addition, it also indicates that the crystalline phases in PP interact with certain crystalline phases in FA and WSP. According to the XRD analysis result, the dominant mineral phases in FA are quartz (Q), anhydrite (A) and lime (L), while in WSP they are quartz (Q), actinolite (a) and nimite (N). Both FA and WSP contain the same quartz mineral and have the highest content, which is consistent with other reported results [26,27]. The XRD analysis results are consistent with the EDS results mentioned above. In addition, with the increase in the content of FA and WSP in the PP/FA/WSP composite materials, the strength of the quartz peak increases significantly. It shows that FA and WSP are fully mixed with PP and the distribution of various substances is uniform.

In Figure 6b, it can be clearly observed that PP100 has nine relatively strong peaks, which appear at 2950, 2917, 2868, 2837, 1455, 1376, 1167, 973 and 842 cm^−1^, respectively. The peaks on 2950, 2917, 2868 and 2837 cm^−1^ may be caused by C–H stretching. The peaks at 1455 and 1376 cm^−1^ may be related to the deformation of CH_2_ and the symmetric deformation of CH_3_, respectively. The peak appearing at 1167 cm^−1^ may be related to the C–C bending on the main chain of PP. The peaks associated with the isotactic polypropylene band appear at 973 and 842 cm^−1^ [28]. The FTIR spectra of FA mainly appear at about 1057, 797, 677 and 452 cm^−1^, which may be assigned asymmetric stretching vibration of Si–O, symmetric stretching vibration of Si–O–Si, symmetric stretching vibration of Si–O–Si or Al–O–Si and bending vibration of Si–O–Si or O–Si–O, respectively [29,30,31]. For WSP, the strong peak appearing near 1000 cm^−1^ may be related to the stretching vibration of Si–O, while other peaks appearing between 400 and 800 cm^−1^ may be attributed to Si–O bending strength vibrations of the SiO_2_. With the addition of FA and WSP, the peaks near 1000 cm^−1^ have been strengthened, especially for PP50 and PP60. This again shows that PP, FA and WSP are uniformly mixed before injection molding.

### 3.2. Mass Variation Characteristics

**Figure 7:**
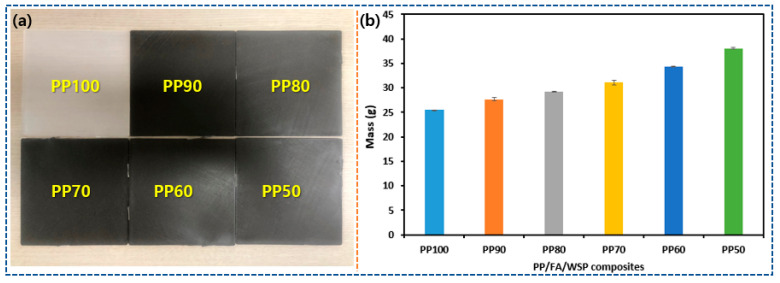
Photograph of PP/FA/WSP composite materials (**a**) and their mass variation (**b**).

Figure 7 shows the mass change characteristics of PP/FA/WSP composite materials with different contents of FA and WSP. The length, width and thickness of all test samples are 100, 100 and 2 mm, respectively. The mass of the sample was measured by a high-precision electronic scale (Model: HS220S, readability: 0.001 g, Hansung Instrument Co., Ltd., Gwangmyeong-si, Gyeonggi-do, Republic of Korea) and each sample was measured three times to calculate its average value. As shown in Figure 7, PP100 has the smallest mass, which is 25.48 g, followed by PP90, PP80, PP70, PP60 and PP50, which are 27.66, 29.26, 31.07, 34.35 and 38.09 g, respectively. With the increase in the mixing amount of FA and WSP, the mass of PP/FA/WSP composite materials presents a linear increase trend. Compared to PP100, the mass of PP90, PP80, PP70, PP60 and PP50 are increased by 8.56%, 14.85%, 21.97%, 34.84 and 49.53%, respectively. This is because the specific gravity of FA and WSP are, respectively, 2.30–2.38 [32] and 2.53–2.80 [33,34], which are both larger than that of PP (0.91) [35]. The mass change characteristics of PP/FA/WSP composite materials provide a reference value for future applications in certain fields.

### 3.3. Thermogravimetric Analysis

**Figure 8:**
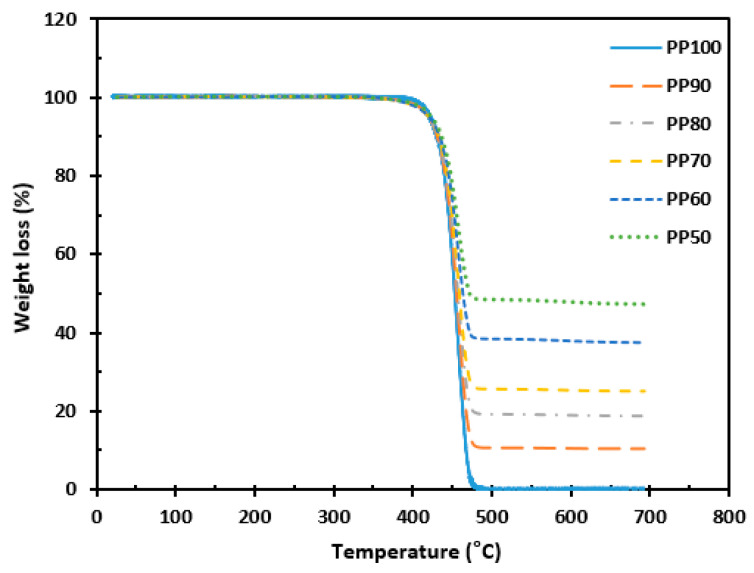
Thermogravimetric analysis for all tested samples.

Figure 8 shows the tensile strength of pure PP and PP/FA/WSP composite materials. As shown in Figure 8, it can be clearly observed that the thermal decomposition of all test samples occurs between 350–500 °C, which is consistent with [36]. Moreover, with the addition of FA and WSP, the thermal decomposition rate of the PP/FA/WSP composite materials is slightly delayed. PP100 is completely decomposed at 500 °C and the remaining amount after decomposition gradually increases with the increase in the amount of FA and WSP added. This is because PP is a high molecular polymer composed of hydrocarbons that has decomposable properties at high temperatures, while FA and WSP are mainly composed of Al_2_O_3_ and SiO_2_ that have nondecomposable properties. In addition, the TGA results also indicate that FA and WSP are highly likely inorganic fillers [37]. When the pyrolysis of the tested samples is completed, the weight loss ratios for PP100, PP90, PP80, PP70, PP60 and PP50 are 99.71%, 89.59%, 81.25%, 74.94%, 62.54% and 52.76%, respectively. The weight loss rate of PP80, PP70, PP60 and PP50 is slightly higher than the expected value, possibly due to the presence of a certain amount of carbon in the FA, which undergoes further oxidation and combustion at high temperatures [38]. Overall, the weight loss rates of PP100, PP90, PP80, PP70, PP60 and PP50 are similar to the ratios of PP to the total amount of FA and WSP, respectively. It indicates that the mixing of PP, FA and WSP is relatively uniform and the preparation of PP/FA/WSP composite materials is successful.

### 3.4. Mechanical Properties

#### 3.4.1. Tensile Strength

**Figure 9:**
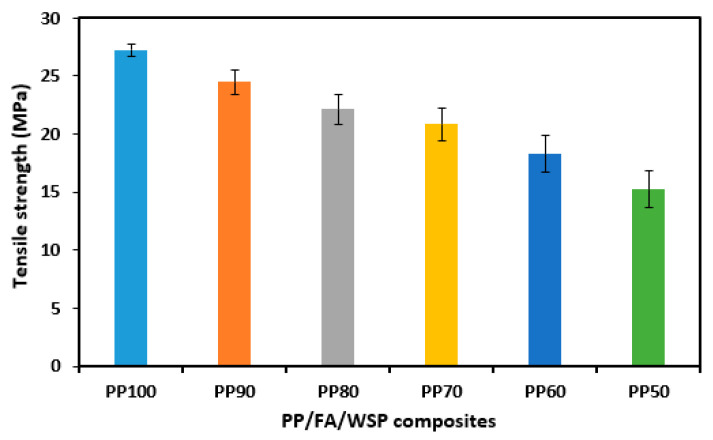
Tensile strength for all tested samples.

Figure 9 shows the tensile strength of pure PP and PP/FA/WSP composite materials. As predicted, the tensile strength gradually decreases with the addition of FA and WSP. The maximum tensile strength of PP100 is 27.24 MPa, followed by PP90, PP80, PP70, PP60 and PP50, which are 24.47, 22.14, 20.85, 18.30 and 15.23 MPa, respectively. Eriksen et al. [37] also reported similar experimental results, with the tensile strength of PP in the range of 27 to 29 MPa and pointed out that its value is the lower limit for virgin PP (21–40 MPa). The lower tensile strength indicates that PP may lead to varying degrees of degradation during mechanical recycling processes [37]. Compared to PP100, the tensile strength of PP90, PP80, PP70, PP60 and PP50 is reduced by 10.15%, 18.72%, 23.47%, 32.83% and 44.08%, respectively. This may be mainly due to the addition of FA and WSP hindering the binding between the carbon chains of PP polymers. Yang et al. [39] also reported a decrease in the tensile strength of PP due to the addition of various lignocellulosic fillers, resulting in a decrease in the interfacial bonding ability of the PP polymer. In addition, Pardo et al. [40] added 30, 60 wt% biomass ash and coupling agents to PP and compared their tensile strength. The research results showed that adding biomass ash would also lead to a decrease in the tensile strength of PP due to the lack of interfacial adhesion between PP and ash. Although the use of coupling agents could improve the tensile strength of ash-containing materials, it was still lower than that of pure PP. However, it is surprising that the addition of biomass ash improved the hardness of PP materials.

#### 3.4.2. Bending Strength

**Figure 10:**
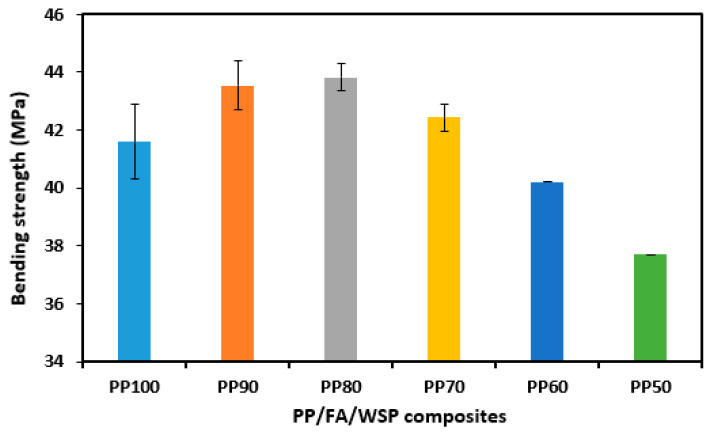
Bending strength for all tested samples.

Figure 10 presents the typical three-point bending strength of PP/FA/WSP composite materials with different FA and WSP contents. Unlike tensile strength, the bending strength of the composite materials exhibits a trend of first increasing and then decreasing with the increase in FA and WSP. The PP/FA/WSP composite material with 10 wt% FA and 10 wt% WSP (PP80) has the maximum bending strength of 43.83 MPa, followed by PP90 of 43.55 MPa, PP70 of 42.43 MPa, PP100 of 41.59 MPa, PP60 of 40.20 MPa and PP50 of 37.69 MPa. Compared to PP100, the bending strength of PP90, PP80 and PP70 is increased by 4.71%, 5.39% and 2.02%, while for PP60 and PP50 it is reduced by 3.34% and 9.38%, respectively. This indicates that adding up to 30 wt% FA and WSP can improve the bending strength of PP to a certain extent, while excessive FA and WSP can lead to a significant decrease in the adhesion between PP molecules, thereby reducing the bending strength. The reason for the increase in bending strength caused by the addition of an appropriate amount of FA and WSP may be attributed to the fact that both FA and WSP contain a large amount of quartz, resulting in an enhanced stress transfer at the interface between FA, WSP and the PP matrix. However, the decrease in bending strength caused by excessive FA and WSP may be due to the reduction in mechanical interlocking between FA, WSP and the PP molecular chain [41]. Therefore, 20 wt% of FA and WSP is the optimal mixing ratio, higher or lower than this results in a decrease in bending strength.

#### 3.4.3. Notched Impact Energy

**Figure 11:**
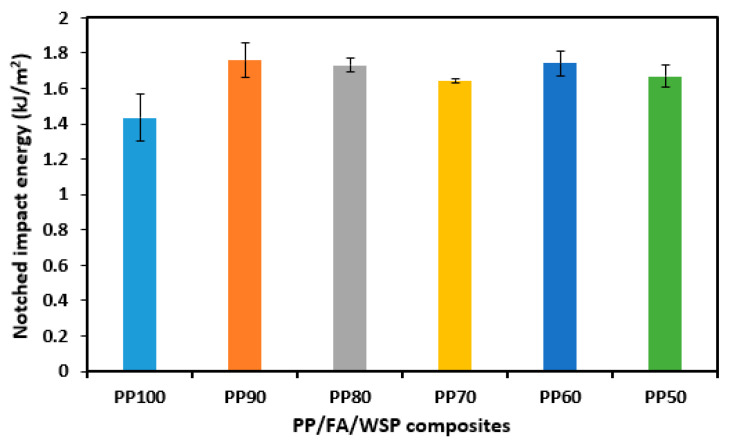
Notched impact energy for all tested samples.

Figure 11 shows the notched impact energy of pure PP and PP/FA/WSP composite materials. As shown in Figure 11, it is clear that the notched impact energy of all PP/FA/WSP composite materials is higher than that of PP100. The PP/FA/WSP composite material with 5 wt% FA and 5 wt% WSP (PP90) has a maximum notched impact energy of 1.76 kJ/m^2^, followed by PP60 of 1.74 kJ/m^2^, PP80 of 1.73 kJ/m^2^, PP50 of 1.67 kJ/m^2^, PP70 of 1.65 kJ/m^2^ and PP100 of 1.44 kJ/m^2^. The notched impact energy of pure PP is similar to that reported by Li et al. [42]. They also pointed out that the large amount of cavitation formed in the elastomer particles can improve the shear yield of the matrix, thereby consuming much more impact energy. Compared to PP100, the notched impact energy of PP90, PP80, PP70, PP60 and PP50 has been improved by 22.22%, 20.14%, 14.58%, 20.83% and 15.97%, respectively. The notched impact energy of the PP/FA/WSP composite materials is between 1.44 and 1.76 kJ/m^2^ and there is no significant change overall. This may be attributed to the abundance of quartz, anhydrite, lime, actinolite and nimite in FA and WSP, which is beneficial for increasing the hardness of the material. In addition, FA and WSP have excellent noncompressibility characteristics, while PP will generate bubbles and pores during high-temperature melting with a certain degree of compressibility. Therefore, the addition of FA and WSP can fill the pores generated between PP to improve impact strength.

## 4. Conclusions

To evaluate the reuse value of various wastes such as waste polypropylene (PP), fly ash (FA) and waste stone powder (WSP), PP, FA and WSP were prepared as PP/FA/WSP composites in different mixing ratios by an injection molding machine. Their characterization, TGA and mechanical properties were investigated. The main findings are as follows:

i. A series of PP/FA/WSP composites were successfully prepared through the injection molding machine, even if the total mixing ratio of FA and WSP is as high as 50 wt%.
ii. SEM, EDS, XRD and FTIR analysis results show that the distribution of FA and WSP in PP is uniform. There are no cracks, fractures or other defects on the surface of PP/FA/WSP composites.
iii. TGA results show that the weight ratio of all PP/FA/WSP composites is similar to the predetermined value. It indicates that FA, WSP and PP are uniformly mixed and the injection molding method is suitable for preparing PP/FA/WSP composite materials.
iv. For mechanical properties, although the addition of FA and WSP leads to a decrease in the tensile strength of PP/FA/WSP composites, the addition of appropriate amounts of FA and WSP leads to an increase in the bending strength and notched impact energy of PP/FA/WSP composites by 2.20–4.71% and 14.58–22.22%, respectively.

## Data Availability

Not applicable.
